# Supplementary material for: Experimental Evolution Reveals Favored Adaptive Routes to Cell Aggregation in Yeast
Source: Genetics. 2017 Apr 21;206(2):1153–67. doi: 10.1534/genetics.116.198895 (PMC5499169; doi:10.1534/genetics.116.198895)

HOPE *et al* SUPPLEMENTARY FIGURES:

Fig. S1: Micrographs at 150X of 23 evolved clones of *S. cerevisiae* with aggregation phenotype. Micrographs received additional processing (grey scale conversion, 20% increase in brightness, 20% increase in contrast) to better highlight the phenotypes. YMD2680 and YMD2689 exhibit mother-daughter separation defects while all other clones exhibit flocculation.

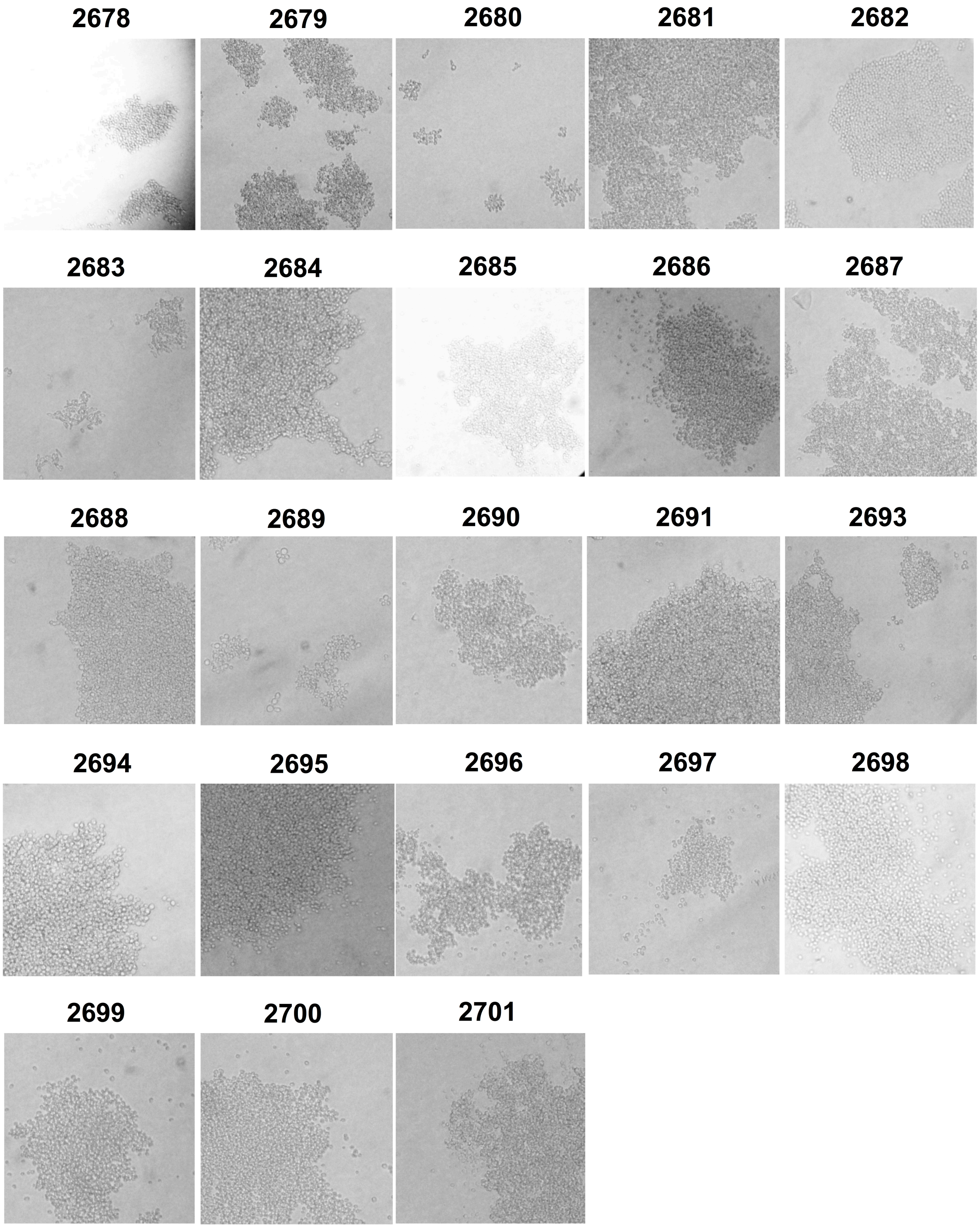

Fig. S2: Mother-daughter separation defects in evolved clones YMD2680 and YMD2689 A) DAPI filter micrographs of evolved clones YMD2680 and 2689 with calcofluor white staining. Staining at bud scars highlights the mother-daughter separation defect in these strains at 630X magnification. B) Micrographs at 150X of null mutants and results of complementation testing by crossing evolved clones to null mutants. Micrographs received additional processing (grey scale conversion, 20% increase in brightness, 20% increase in contrast) and are cropped to better highlight the phenotypes.

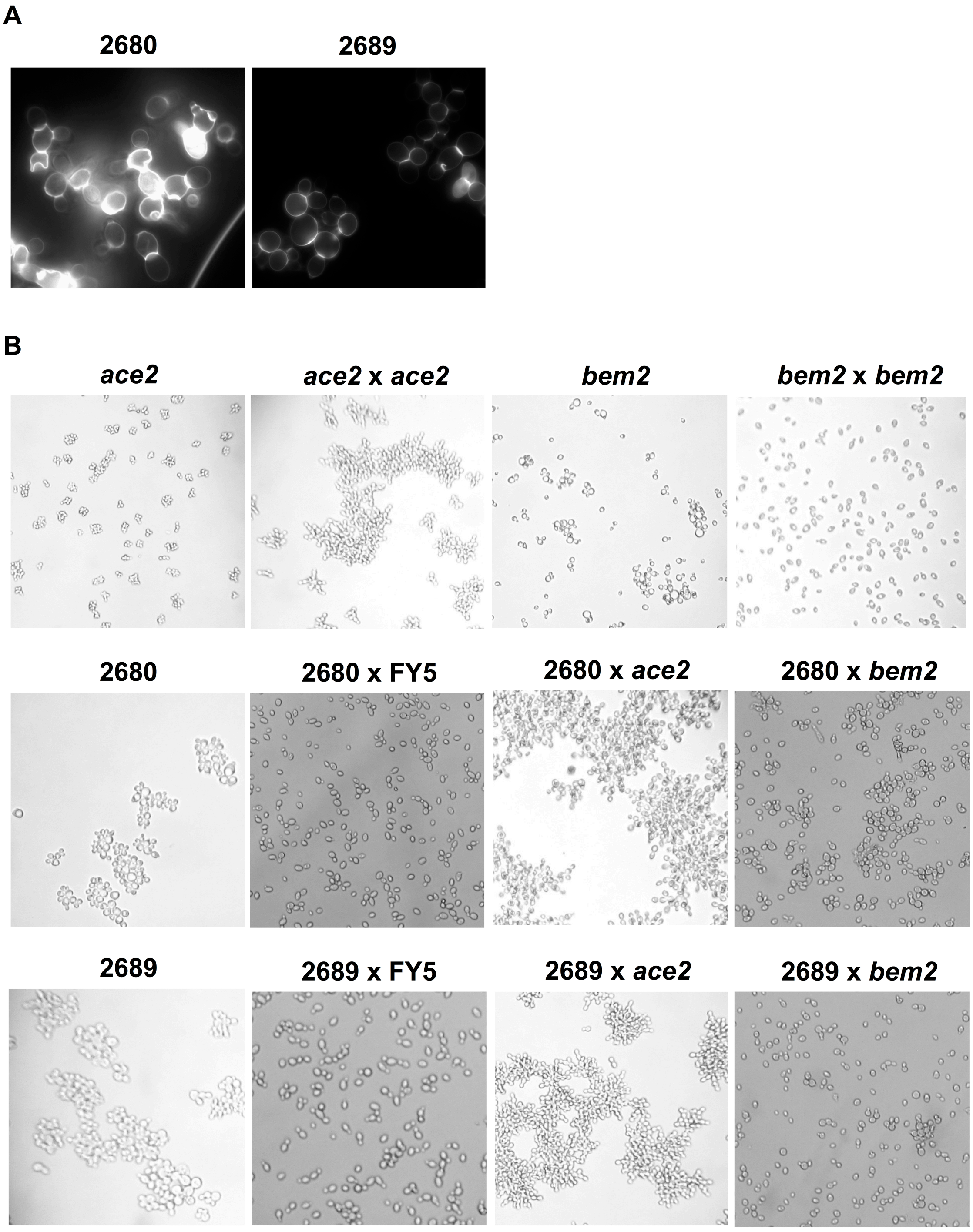

Fig. S3: EDTA treatment of evolved clones stratifies mother-daughter separation from true flocculation. Micrographs received additional processing (grey scale conversion, 20% increase in brightness, 20% increase in contrast) to better highlight the phenotypes. Aggregates caused by separation defects fail to disperse following treatment with 4mM EDTA.

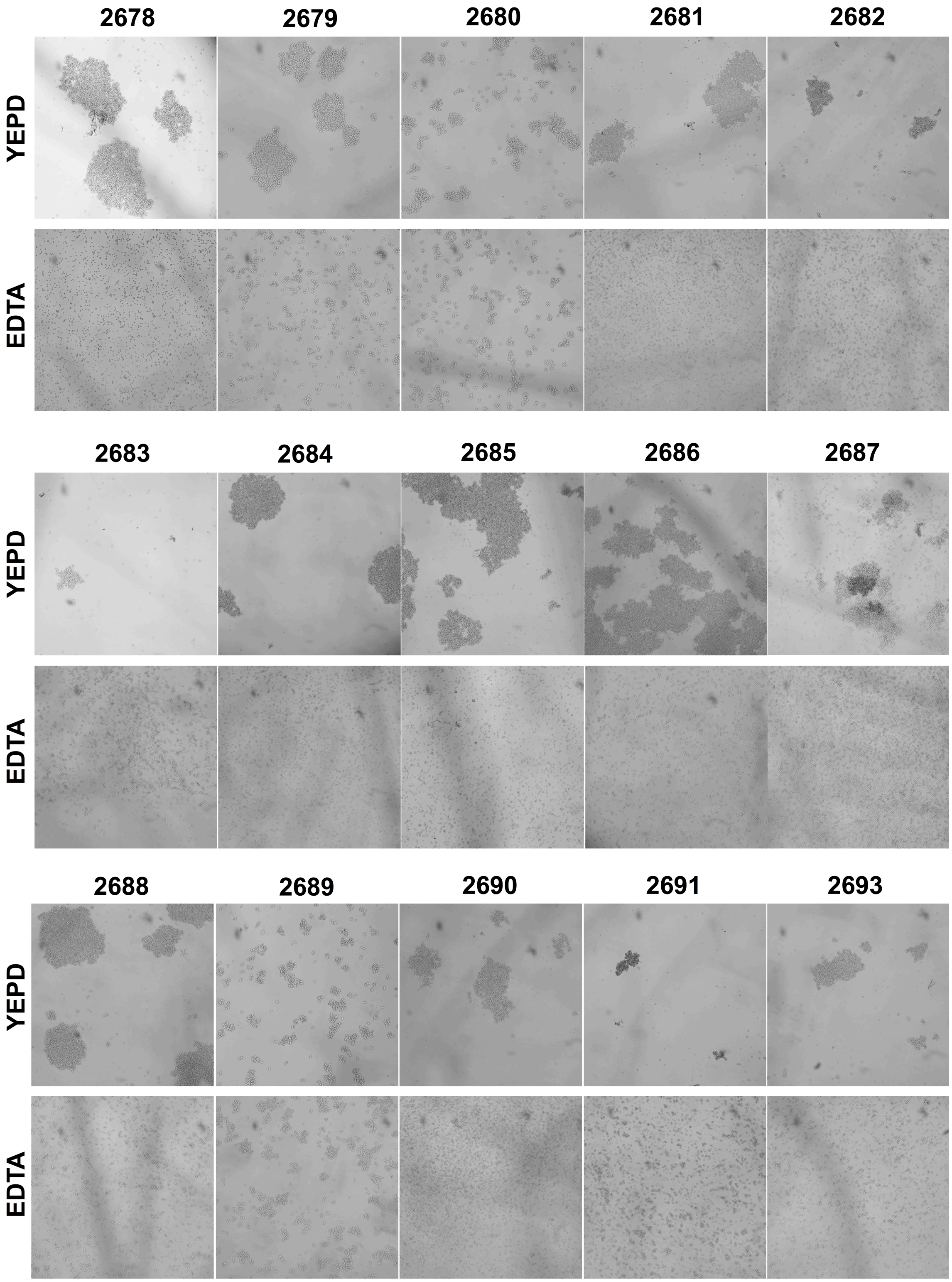

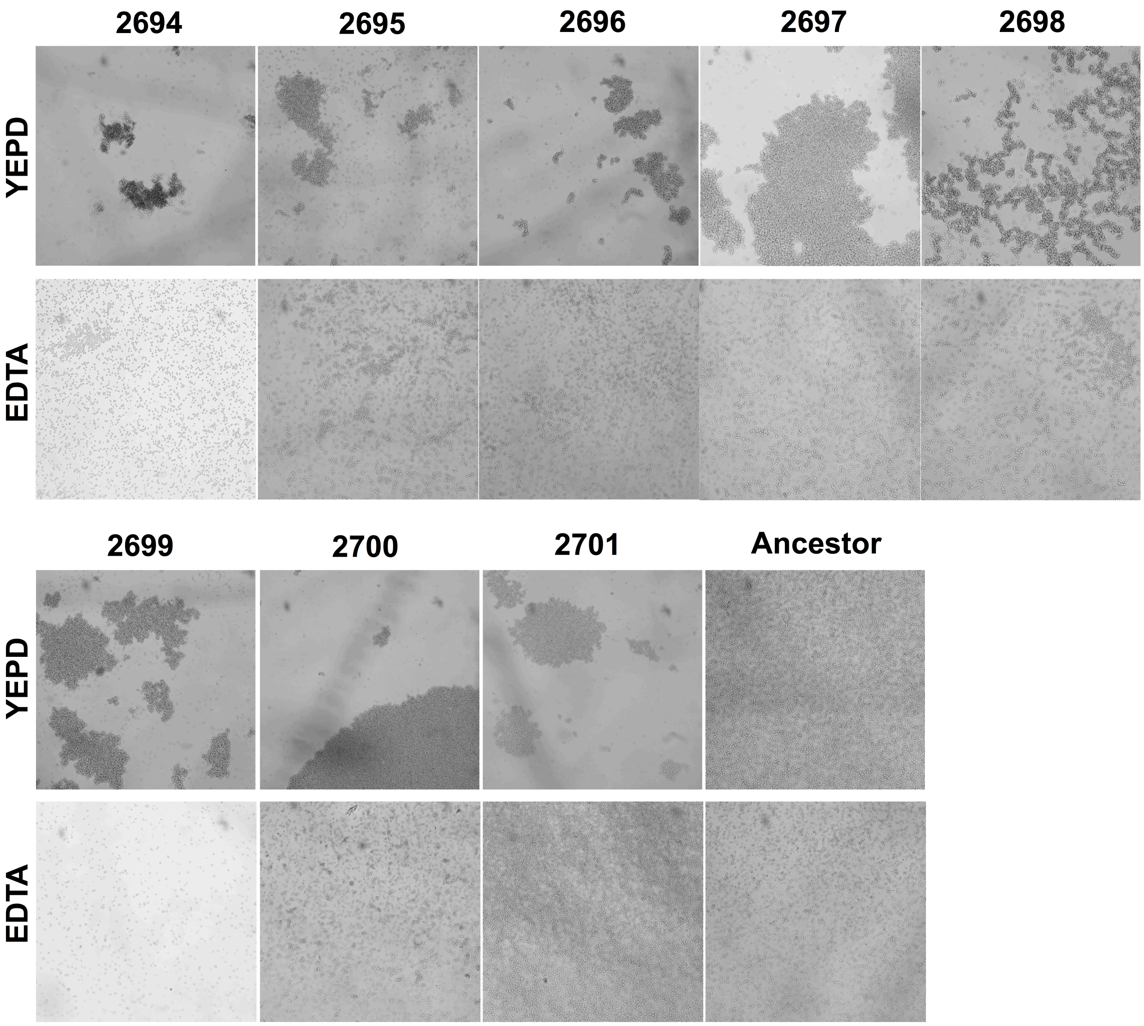
2694
2695
2696
2697
2698
YEPD
EDTA
2699
2700
2701
Ancestor
YEPD
EDTA

Fig. S4: Settling ratios harbor quantitative variation, particularly among clones with Ty insertions in the *FLO1* promoter. Distribution of settling ratios among a subset of settling segregants from the backcross performed with each evolved clone. Data for each clone is arranged with the top panel displaying data for clones without the *FLO1* promoter insertion, and the bottom panel for strains with the promoter insertion. Variation among segregants provides evidence of additional genetic modifiers of the phenotype beyond the single causal allele identified through BSA and WGS. Clone YMD2683 has a verified secondary modifier and a clear distribution of settling segregants into two groups. Horizontal lines represent independent biological replicates for the evolved clone contributing to each cross, each a mean of three measurement replicates.

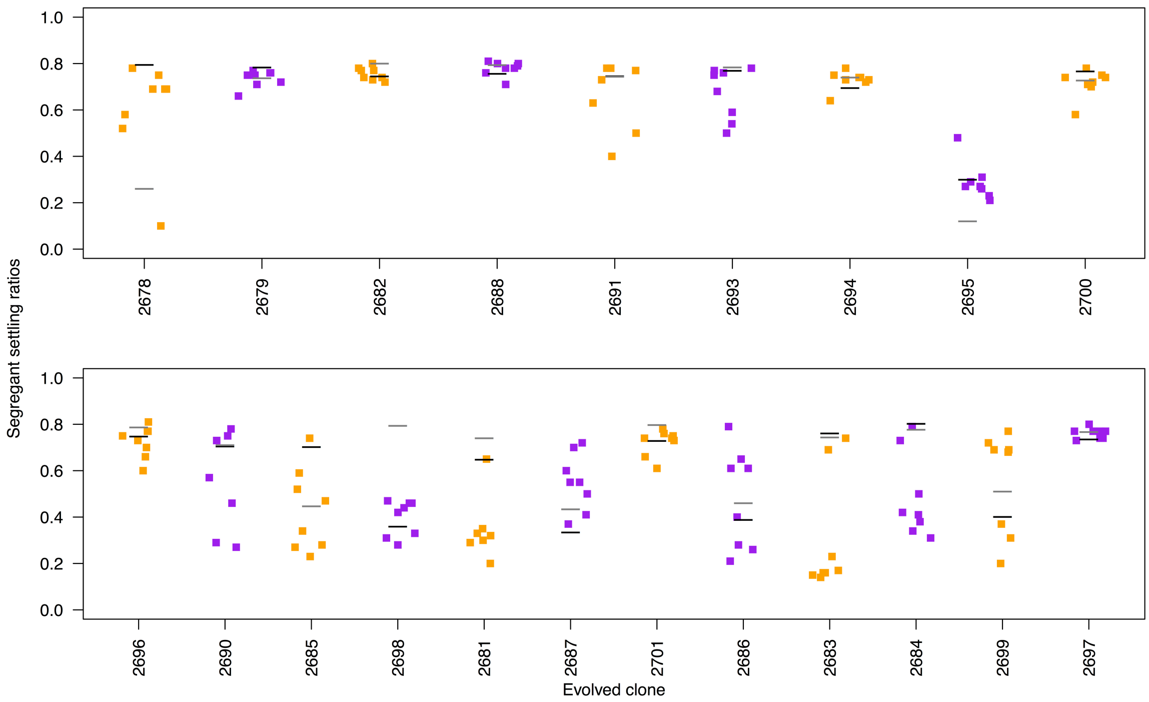

Fig. S5: Segregation of settling trait in a backcross between evolved clone YMD2701 and lab strain FY5 is explained by chromosome I aneuploidy. A) In this cross, settling trait does not segregate 2:2. 16 representative tetrads are shown horizontally with segregation patterns of 2:2, 1:3, and 0:4 non-settling:settling. **B)** Copy number analysis of clone YMD2701 reveals a chromosome I amplification. Y-axis values represent relative copy number increase, from 1 copy lost (0) to 3 copies gained (4) from a haploid starting point. X-axis values are genome coordinate. Gene *FLO1* is on chromosome I, and clone YMD2701 is known to have a Ty insertion in the *FLO1* promoter. **C)** Three possible outcomes of meiosis with the causal *FLO1* Ty insertion on both copies of chromosome I leads to the observed segregation pattern.

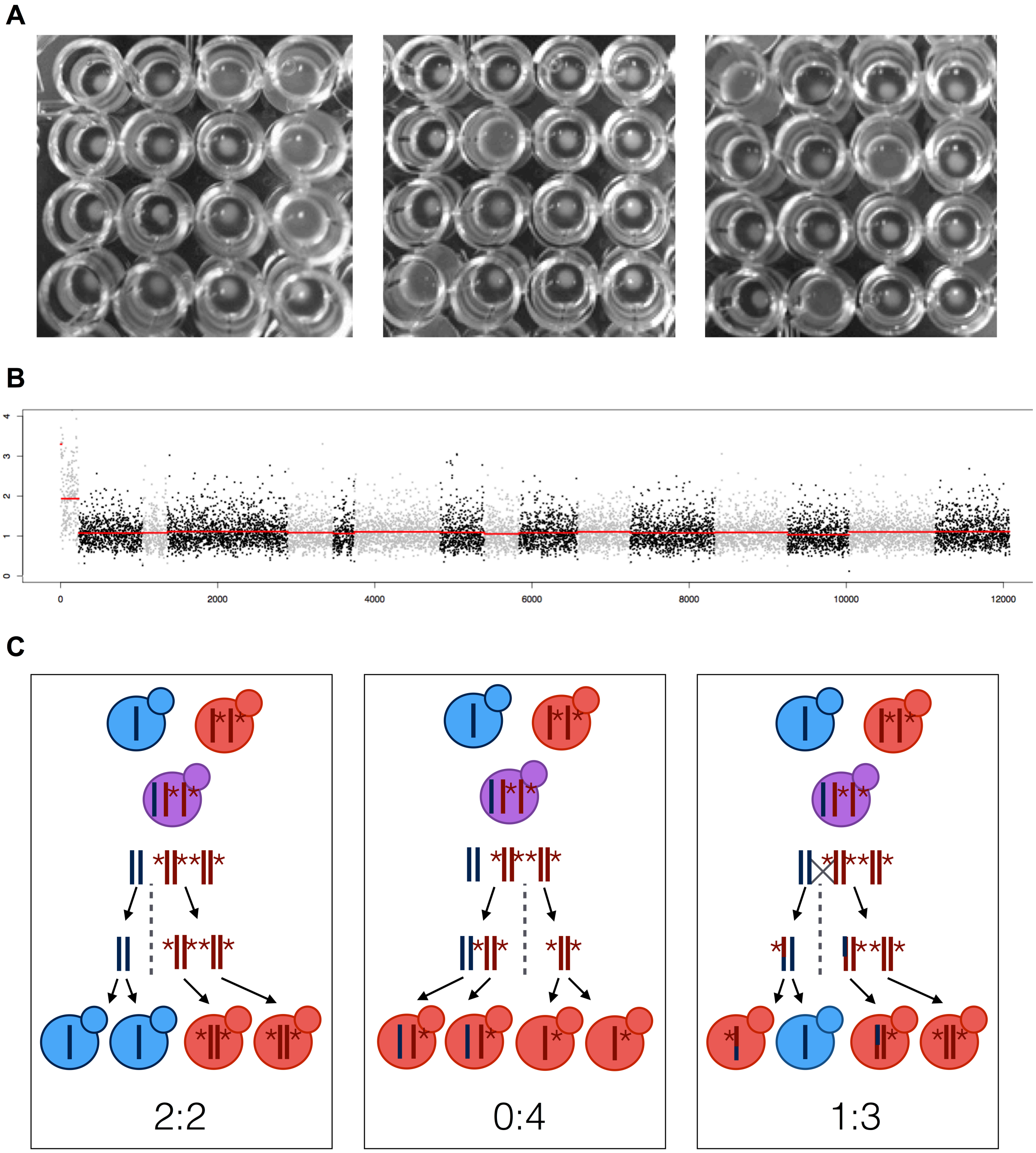

Fig. S6: Micrographs of evolved populations of wild-type and *flo1* knockout *S. cerevisiae* strains from the final experiment time point of the knockout evolution. Images are arranged to match the experimental setup in four 30°C heat blocks, A-D, receiving identical treatment. Red boxes surround images for vessels with a visible aggregation trait at the final time point, blue boxes surround images for vessels with mother-daughter separation defects recorded via microscopy, and a purple box surrounds the image for a vessel with a visible aggregation phenotype and possible separation defect. Vessels 1-4 and 9-12 in each block were inoculated with the wild-type strain, and vessels 5-8 and 13-16 contain the knockout strain. Missing images are for populations that were lost to contamination prior to the final time point. Micrographs received additional processing (grey scale conversion, 20% increase in brightness, 20% increase in contrast) to better highlight the phenotypes.

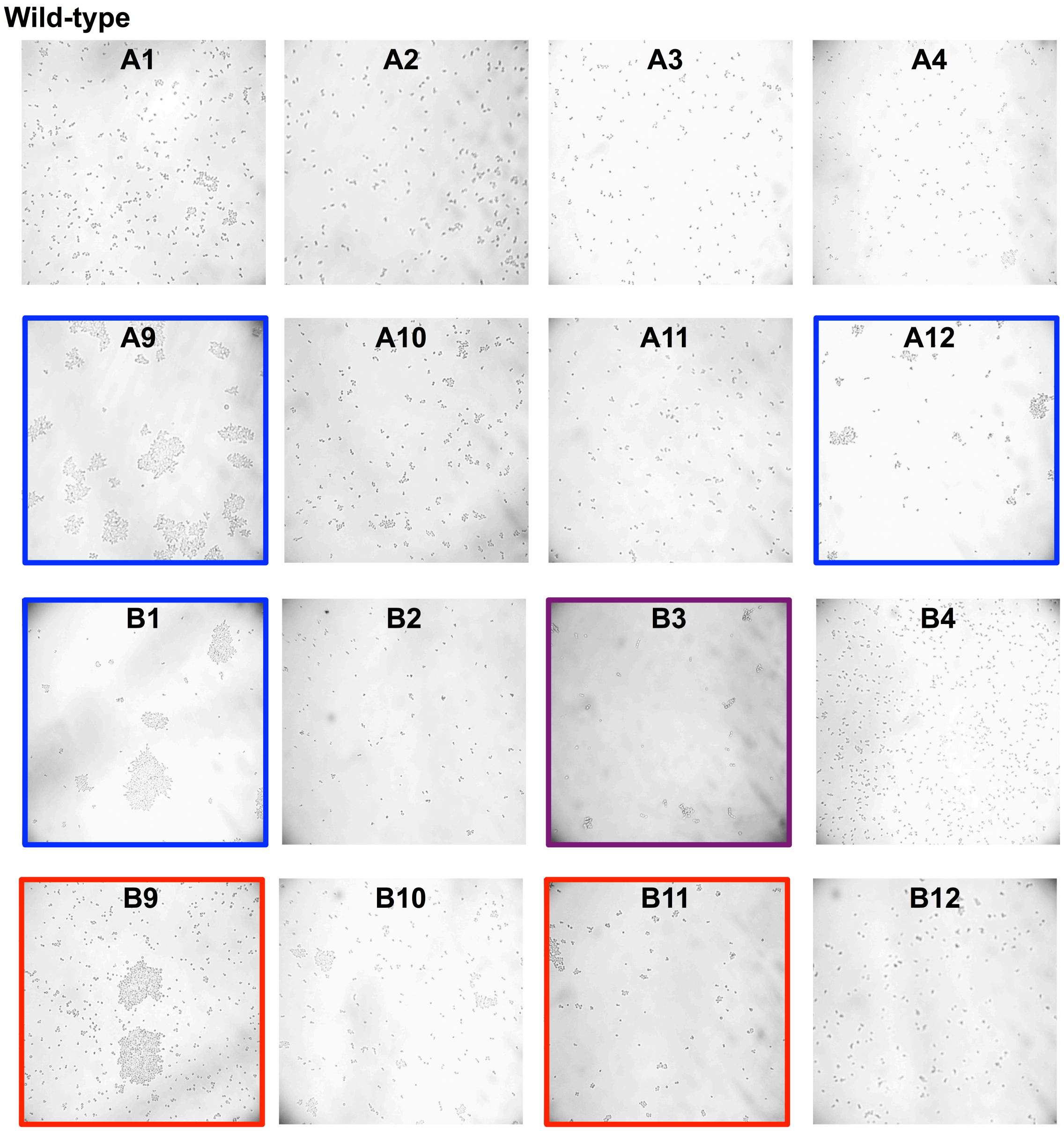

Wild-type

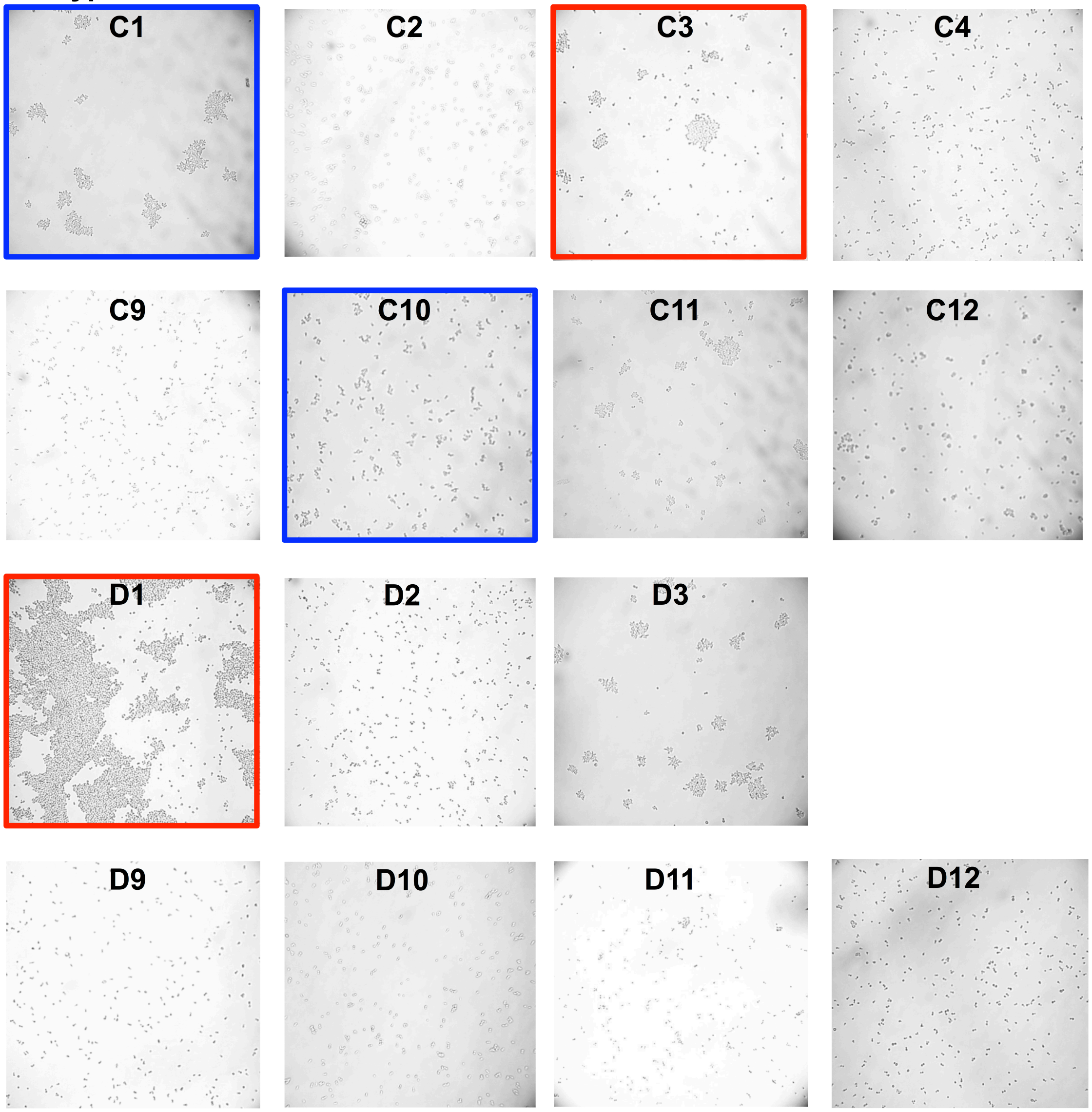

***flo1* knockout**

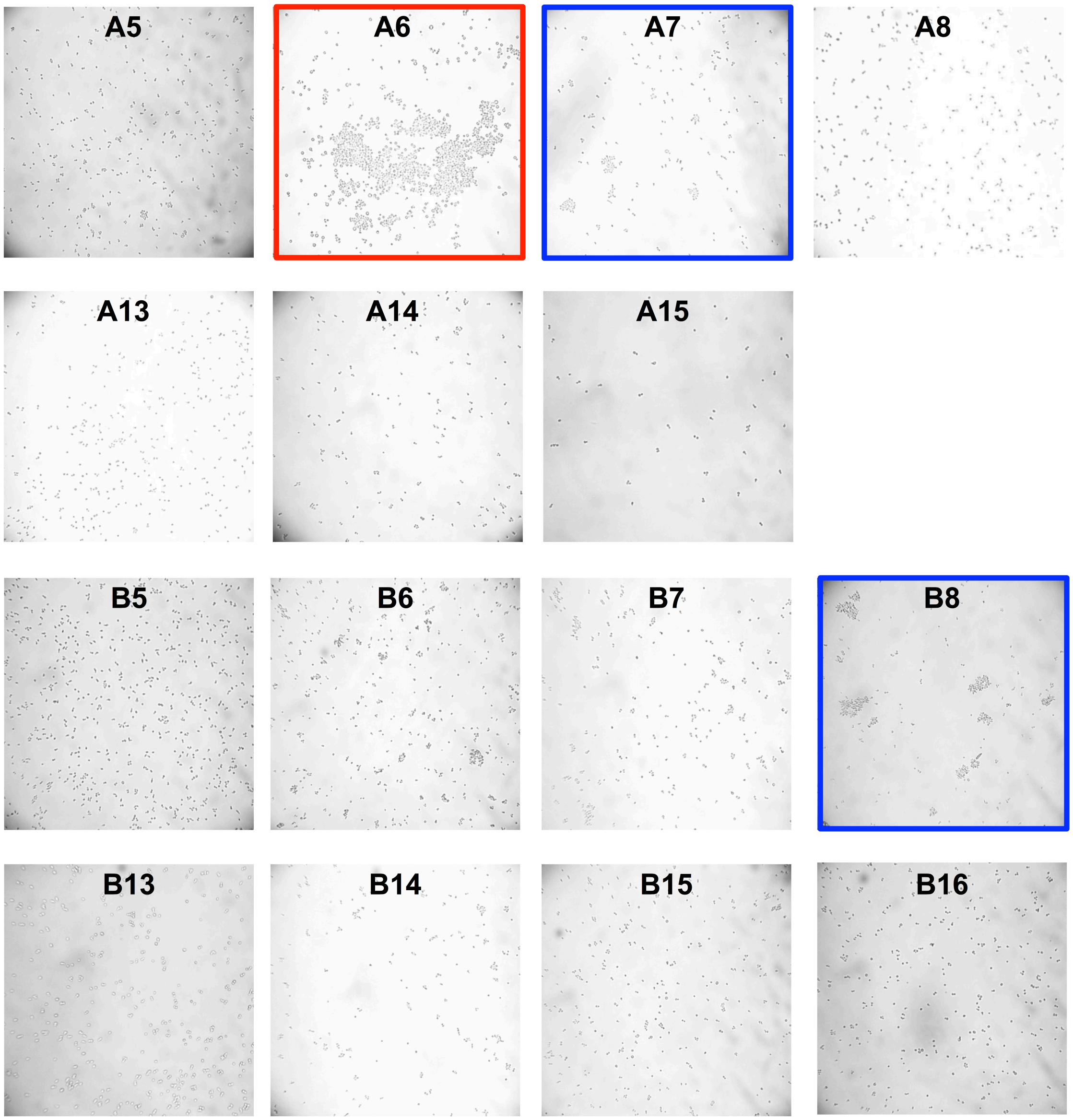

***flo1* knockout**

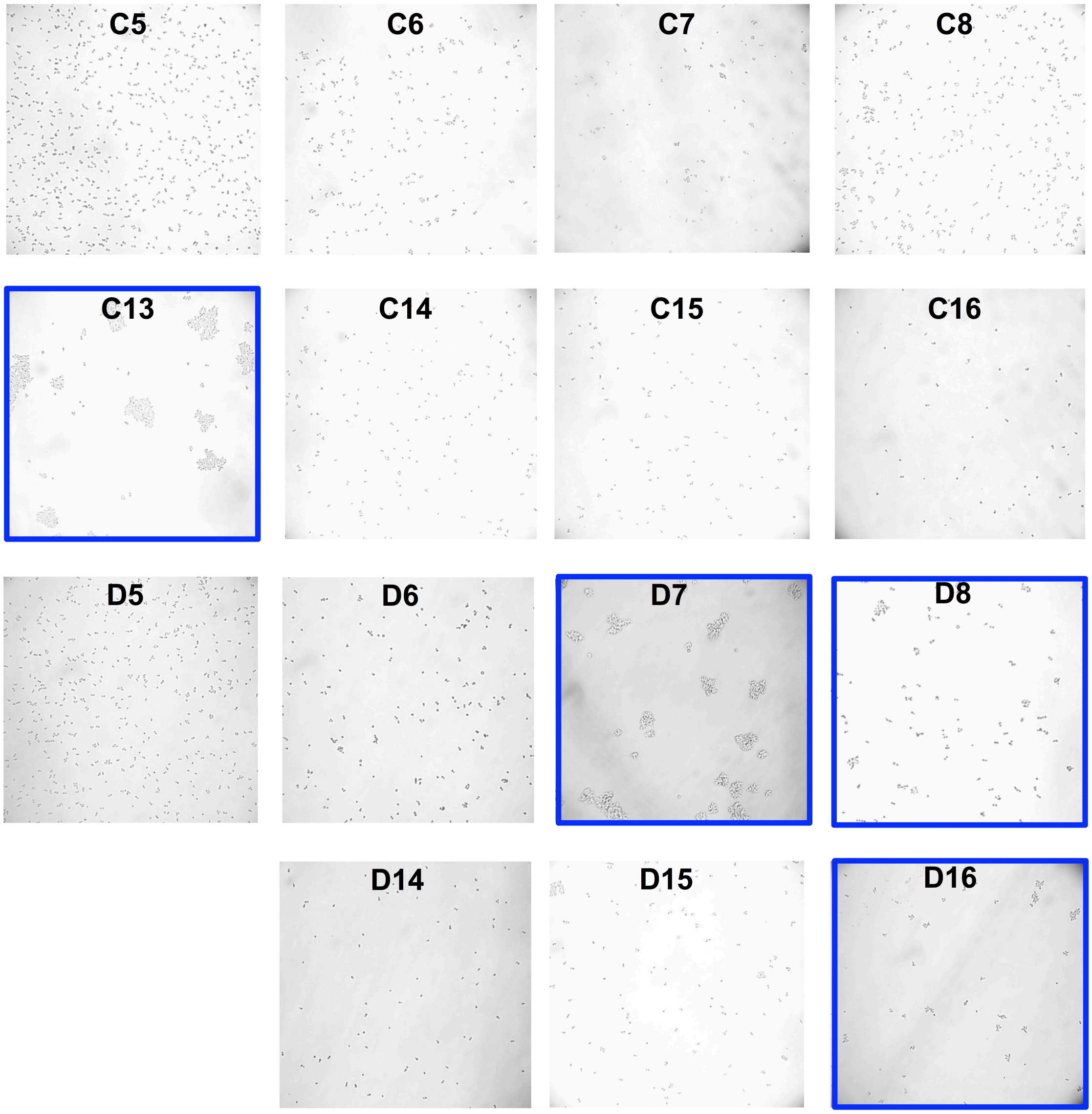

Supplement: Supplementary file 1 [file 1153FileS1.pdf]
