# Supplementary material for: Experimental Evolution Reveals Favored Adaptive Routes to Cell Aggregation in Yeast
Source: Genetics. 2017 Apr 21;206(2):1153–67. doi: 10.1534/genetics.116.198895 (PMC5499169; doi:10.1534/genetics.116.198895)
Supplement: Supplementary file 2 [file 1153FileS2.pdf]

HOPE *et al* SUPPLEMENTARY TABLES:

Table S1: Raw quantitative measurements for settling ratios. Data visualized in main text Figure 1 and Supplementary Figure 4, provided as separate Excel file.

Tab 1: Clone_data_rep1. Three measurement replicates from ImageJ (columns B-H) are provided for the 60-minute settling image for each evolved clone, with a calculation of the coordinate of half of the maximum grey value (I) as in (Hope and Dunham 2014), and a final calculation of the settling ratio (J).

Tab 2: Clone_data_rep2. A second biological replicate for each original clone, with three measurement replicates per image.

Tab 3: Segregant_data. A single measurement and ratio is provided for multiple progeny from the backcross of each original evolved clone (numbers in column A) to the laboratory strain FY4.

Table S2: List of high quality mutations called for each evolved clone from whole genome sequencing. Provided as separate Excel file. Raw sequencing data is available under NCBI BioProject PRJNA339148, BioSample accessions SAMN05729740-5729793 and filtering was performed as described in Materials and Methods, with additional filtering steps detailed below. Mutations verified as causal have been highlighted in green. This table lists high quality SNPs (Tab 1), indels (Tab 1), Ty element insertions (Tab 2), structural variants (SVs) (Tab 3), and copy number variants (CNVs) (Tab 4) for all evolved clones. Tabs 1-3 have the following column labels: Sample: sample number (note "EV" samples are from second evolution experiment); Chrom: chromosome; Pos: chromosome location; Ref: reference allele; Alt: alternative allele; Qual: quality score; Info: descriptive mutation information generated by bcftools, retroseq, or lumpy; Format: format of genotype information; Genotype: genotype information generated by bcftools, retroseq, or lumpy; Mutation type: coding-nonsynonynous, coding-synonymous, intergenic, or 5'-upstream; Gene: systematic gene name; AA: amino acid annotation; Gene Alias: common gene name. Tab 4 has the following additional column labels: Start: start location of copy number segment; End: end location of copy number segment; Copy Number: average copy number for segment, calculated using DNAcopy.

Tab 1: SNPs_indels. SNPs/indels present in 7 or more samples for the original 23 evolved clones or 4 or more samples for the 5 new evolved clones were filtered out to remove common false positives or ancestral mutations, using bcftools isec (Li and Durbin 2009; Faust and Hall 2014). Mutations were then filtered for quality (QUAL>50, DP>=10), and mutations annotated as telomeric, mitochondrial, LTR_retrotransposon, intergenic, or coding-synonymous were removed. All remaining mutations were visually confirmed in IGV. See bctools documentation for genotype and quality annotation information.

Tab 2: retroseq. Ty insertions were called using the program retroseq (Keane, Wong, and Adams 2013) with non-default paramaters (discover: -q 28 -id 85 -len 25 -align, call: -depth 400), annotated (97), and verified by visual inspection in IGV. Intergenic mutations were ignored, excepting *FLO* gene promoters, which were manually re-annotated as 5'-upstream mutations. Additionally, known Ty insertions in the *FLO1* promoter were not called by retroseq in Samples 2, 4 and 8, though PCR and visual inspection indicate otherwise. Note that retroseq gives inexact breakpoints so insertion positions are approximate. See retroseq documentation for genotype and quality annotation information.

Tab 3: lumpy. SVs were called using the program lumpy (Layer et al. 2014) with default paramaters. SVs with at least 10 supporting reads were confirmed using visual inspection in IGV. See lumpy documentation for information on SV type and quality scores.

Tab 4: CNV. CNVs were called as described in Material and Methods using the R program DNAcopy (Seshan and Olshen 2015), with additional visual inspection to validate the findings. To remove noise that would otherwise cause unnecessary splits, a standard deviation correction of 2 was implemented. Chromosomes or chromosome segments that had a copy number different from 1 are listed.

Table S3: Aggregation candidate genes. Curated list of candidate genes with known contributions to separation defects, flocculation, or other biofilm-related phenotypes and their publication(s) of origin.

Gene	Systematic Name	Study
ACE2	*YLR131C*	Ratcliff 2015
AGA1	*YNR044W*	Brückner and Mösch 2012
AMN1	*YBR158W*	Li 2013
ASH1	*YKL185W*	Brückner and Mösch 2012
CDC28	*YBR160W*	Lee 2011
CYC8	*YBR112C*	Brückner and Mösch 2012
CYR1	*YJL005W*	Granek 2013
DEP1	*YAL013W*	Brückner and Mösch 2012
DIA1	*YMR316W*	Palecek 2000
END3	*YNL084C*	Taylor 2014
FIG2	*YCR089W*	Brückner and Mösch 2012
FLO1	*YAR050W*	Brem 2002
FLO10	*YKR102W*	Brückner and Mösch 2012
FLO11	*YIR019C*	Granek 2013, Ryan 2012
FLO1p	*YAR062W*	Vestrepen 2005
FLO5	*YHR211W*	Brückner and Mösch 2012
FLO8	*YER109C*	Granek 2013, Brem 2002, Taylor 2014, Ryan 2012
FLO9	*YAL063C*	Roop 2013
FUS3	*YBL016W*	Brückner and Mösch 2012
GCN1	*YGL195W*	Granek 2013
GCN2	*YDR283C*	Brückner and Mösch 2012
GCN4	*YEL009C*	Brückner and Mösch 2012
GLN3	*YER040W*	Brückner and Mösch 2012
GPB1	*YOR371C*	Taylor 2016
HAA1	*YPR008W*	Brückner and Mösch 2012
HDA1	*YNL021W*	Brückner and Mösch 2012
HOG1	*YLR113W*	Cullen 2015
HOT1	*YMR172W*	Granek 2013
IRA1	*YBR140C*	Roop 2013, Taylor 2016
IRA2	*YOL081W*	Roop 2013, Taylor 2014, Taylor 2016
IRC8	*YJL051W*	Taylor 2016
KSS1	*YGR040W*	Brückner and Mösch 2012
MFG1	*YDL233W*	Ryan 2012
MGA1	*YGR249W*	Borneman 2006, Brückner and Mösch 2012
MSB2	*YGR014W*	Brückner and Mösch 2012
MSN2	YMR037C	Granek 2013
MSS11	YMR164C	Su 2009, Kim 2004, Kim 2014, Taylor 2014, Ryan 2012
NRG1	YDR043C	Brückner and Mösch 2012

Gene	Systematic Name	Study
NRG2	*YBR066C*	Brückner and Mösch 2012
PGU1	*YJR153W*	Cullen 2015
PHD1	*YKL043W*	Borneman 2006, Brückner and Mösch 2012
PPM1	*YDR435C*	Granek 2013
PRP42	*YDR235W*	Granek 2013
RGA1	*YOR127W*	Li 2013
RGT1	*YKL038W*	Granek 2013
RIM101	*YHL027W*	Brückner and Mösch 2012
RME1	*YGR044C*	Brückner and Mösch 2012
SFL1	*YOR140W*	Brückner and Mösch 2012, Taylor 2016
SKS1	*YPL026C*	Granek 2013
SLF1	*YDR515W*	Granek 2013
SNF1	*YDR477W*	Bruckner and Mosch 2012
SOK2	*YMR016C*	Borneman 2006, Brückner and Mösch 2012
SOL3	*YHR163W*	Granek 2013
SSN3	*YPL042C*	Taylor 2016
SSN8	*YNL025C*	Taylor 2016
STA1	*STA1*	Kim 2004, Kim 2014
STE12	*YHR084W*	Kim 2004, Brückner and Mösch 2012
SWI5	*YDR146C*	Brückner and Mösch 2012
TEC1	*YBR083W*	Kim 2004, Brückner and Mösch 2012
TPK1	*YJL164C*	Brückner and Mösch 2012
TPK2	*YPL203W*	Brückner and Mösch 2012
TRR1	*YDR353W*	Taylor 2014
TUP1	*YCR084C*	Brückner and Mösch 2012
URE2	*YNL229C*	Brückner and Mösch 2012
YAK1	*YJL141C*	Granek 2013, Brückner and Mösch 2012
YAP1	*YML007W*	Brückner and Mösch 2012

Table S4: Primers used in this study

Primer	Sequence 5'-3'	Amplicon size	Experiment
EH030PF	CAATATGCAAGCTCCTGGCA	2.2kb	Amplifies *FLO11* repeats from S288C - matches Up776flo11 primer from Zara 2009
EH030PR	GCCAGGGTATTTGGATGATG	2.2kb	pair for EH030PF
EH045PF	GAATTGTGCGGACGTTCCTC	507bp	Amplifies *HSL7* around potential secondary modifier mutation for YMD2683
EH045PR	GTGGAGGCGCCAATATTAGC	507bp	pair for EH045PF
EH046PF	CTGGCAGCGCTACTATCTCA	676bp	Amplifies *IRA1* around potential secondary modifier mutation for YMD2683
EH046PR	GCATTCACACTCGACTGCTT	676bp	pair for EH046PF
EH047PF	GCTACCTGCAATTGCATCAC	533bp	Amplifies *VTS1* around potential secondary modifier mutation for YMD2683
EH047PR	GACCAGCATTAGGATGCGTA	533bp	pair for EH047PF
EH048PF	GAGAGGCCACTGAGAGAGTA	596bp	Amplifies *TCP1* around potential secondary modifier mutation for YMD2683
EH048PR	CAGAGTCAGCACCAATGATC	596bp	pair for EH048PF
CJA007F	TCCACGGAGACATACGTTTG	2.1kb/8.1kb	Amplifies promoter region of *FLO1* to identify Ty insertions; 2.1kb without Ty; 8.1kb with Ty
CJA007R	TGTCCTCCGACAGAACCTAG	2.1kb/8.1kb	pair for CJA007F
CJA009F	TATTCGGAAGGCATGATGTC	2.5kb	Validates correct insertion of *FLO1* into S288C genome for knockout construction
CJA009R	TAAGCGAACCACACTAGATC	2.5kb	pair for CJA009F
EH052PF	GCTCATCCTTATTCGGCTCC	391bp	Amplifies *HOG1* around potential secondary modifier mutation for YMD2690
EH052PR	GTATGGCCTGGTTACCGTAG	391bp	pair for EH052PF
EH053PF	CTACAGCTCCTTATCCGGTG	425bp	Amplifies *MIT1* around deletion to confirm breakpoints for YMD2694
EH053PR	ATTGTTCGCGTGACCCATAG	425bp	pair for EH053PF
EH054PF	ATCTTGTTCTGGATGAGGCC	6.4kb	Amplifies *YBLWTy2-1* from S288C genome
EH054PR	CAAGAGGGAGCCGCTATTTC	6.4kb	pair for EH054PF
